# Phenolics from *Garcinia mangostana* Inhibit Advanced Glycation Endproducts Formation: Effect on Amadori Products, Cross-Linked Structures and Protein Thiols

**DOI:** 10.3390/molecules21020251

**Published:** 2016-02-22

**Authors:** Hossam M. Abdallah, Hany El-Bassossy, Gamal A. Mohamed, Ali M. El-Halawany, Khalid Z. Alshali, Zainy M. Banjar

**Affiliations:** 1 Department of Natural Products, Faculty of Pharmacy, King Abdulaziz University, Jeddah 21589, Saudi Arabia; gamals2001@yahoo.com (G.A.M.); ahalawany2003@yahoo.com (A.M.E.H.); 2 Department of Pharmacognosy, Faculty of Pharmacy, Cairo University, Cairo 11562, Egypt; 3 Department of Pharmacology, Faculty of Pharmacy, King Abdulaziz University, Jeddah 21589, Saudi Arabia; helbassossy@kau.edu.sa; 4 Department of Pharmacology, Faculty of Pharmacy, Zagazig University, Zagazig 44519, Egypt; 5 Pharmacognosy Department, Faculty of Pharmacy, Al-Azhar University, Assiut Branch, Assiut 71524, Egypt; 6 Departement of Medicine, Faculty of Medicine, King Abdulaziz University, Jeddah 21589, Saudi Arabia; kzalshal@yahoo.com; 7 Departement of Clinical Biochemistry, Faculty of Medicine, King Abdulaziz University, Jeddah 21589, Saudi Arabia; zmbanjar@yahoo.com

**Keywords:** advanced glycation endproducts, mangosteen, amadori product, epicatechin, benzophenone, garcimangosone D, aromadendrin

## Abstract

Accumulation of Advanced Glycation Endproducts (AGEs) in body tissues plays a major role in the development of diabetic complications. Here, the inhibitory effect of bioactive metabolites isolated from fruit hulls of *Garcinia mangostana* on AGE formation was investigated through bio-guided approach using aminoguanidine (AG) as a positive control. Including *G. mangostana* total methanol extract (GMT) in the reaction mixture of bovine serum albumin (BSA) and glucose or ribose inhibited the fluorescent and non-fluorescent AGEs formation in a dose dependent manner. The bioassay guided fractionation of GMT revealed isolation of four bioactive constituents from the bioactive fraction; which were identified as: garcimangosone D (**1**), aromadendrin-8-*C*-glucopyranoside (**2**), epicatechin (**3**), and 2,3′,4,5′,6-pentahydroxybenzophenone (**4**). All the tested compounds significantly inhibited fluorescent and non-fluorescent AGEs formation in a dose dependent manner whereas compound **3** (epicatechin) was found to be the most potent. In search for the level of action, addition of GMT, and compounds **2**–**4** inhibited fructosamine (Amadori product) and protein aggregation formation in both glucose and ribose. To explore the mechanism of action, it was found that addition of GMT and only compound (**3**) to reaction mixture increased protein thiol in both glucose and ribose while compounds **1**, **2** and **4** only increased thiol in case of ribose. In conclusion, phenolic compounds **1**–**4** inhibited AGEs formation at the levels of Amadori product and protein aggregation formation through saving protein thiol.

## 1. Introduction

On a long term basis, the diabetic’s health condition is complicated by repeated blood glucose level elevations. During hyperglycemia, the carbonyl groups of glucose react non-enzymatically with proteins resulting in the formation of structures called Schiff’s bases. This is followed by rearrangement to produce Amadori products like fructosamine [1]. Later, Amadori products form cross-linked structures termed Advanced Glycation Endproducts (AGEs) [2] which play an important role in the development of chronic diabetic complications such as retinopathy, nephropathy, neuropathy, age-related diseases, atherosclerosis and Alzheimer’s disease [2,3,4,5,6]. AGEs can be classified into two major groups: fluorescent crosslinking structures (pentosidine, crosslines, and imidazolones) and non-fluorescent non-crosslinking structures (*N*_ε_-carboxymethyl lysine, *N*_ε_-CML) [7]. Moreover, several AGEs activate the cell surface AGE receptor (RAGE) to initiate -NF-κB and associated pro-inflammatory signaling pathways [8]. Despite significant developments in antidiabetic therapy, most of the antidiabetic drugs available in the market today act mainly to control blood glucose levels, but even if normoglycemia is achieved, diabetics still display a higher risk of developing complications, suggesting a real need for additional treatments to prevent diabetic complications [9]. Herbal drugs are widely used all over the world due to their high efficacy, fewer side effects and relatively low cost. In this regard, some plant extracts have been evaluated for their effects on the formation of AGEs [10,11,12,13]. It is noted that the inhibitory effects of these plant extracts on the formation of AGEs are mainly contributed by the large amount of phenolic antioxidants they contain. As free radicals are involved in the formation of AGEs, it is reasonable to assume that phenolic antioxidants can inhibit the formation of AGEs [14].

Mangosteen (*Garcinia mangostana* Linn., Clusiaceae) is a tropical evergreen tree known for producing a delicious fruit that used traditionally in Indonesia to prevent ulcers, diarrhea, fever, hypertension, obesity and diabetes mellitus. Moreover, it shows antidiabetic effects through α-glucosidase inhibition [15]. Additionally, it was reported that mangosteen showed strong vasorelaxant activity on isolated rat aorta [16]. Recently, the inhibitory effect of *G. mangostana* L. (GMT) on the formation of pentosidine, one of the AGEs, and the remedial effect on skin conditions were measured [17]. Phytochemical investigation of *G. mangostana* proved the presence of different phenolic constituents, including oxygenated and prenylated xanthones, benzophenones, flavonoids and anthocyanins [18,19]. 

The aim of the current study is to evaluate the inhibitory effect of methanol extract of fruit hulls of *G. mangostana* L. (GMT) on AGEs formation, to identify the bioactive fraction and metabolites and illustrate the possible site(s) and mechanism(s) of action. The inhibitory effect of GMT on AGE formation was assessed through determination of fluorescent and non-fluorescent AGEs as well as studing the mechanism of action through measuring Amadori product and protein aggregation formation and its ability to save protein thiols.

## 2. Results and Discussion

### 2.1. Effect of GMT, Bioactive Fraction and Active Metabolites on Advanced Glycation End-Products (AGEs) 

**Figure 1:**
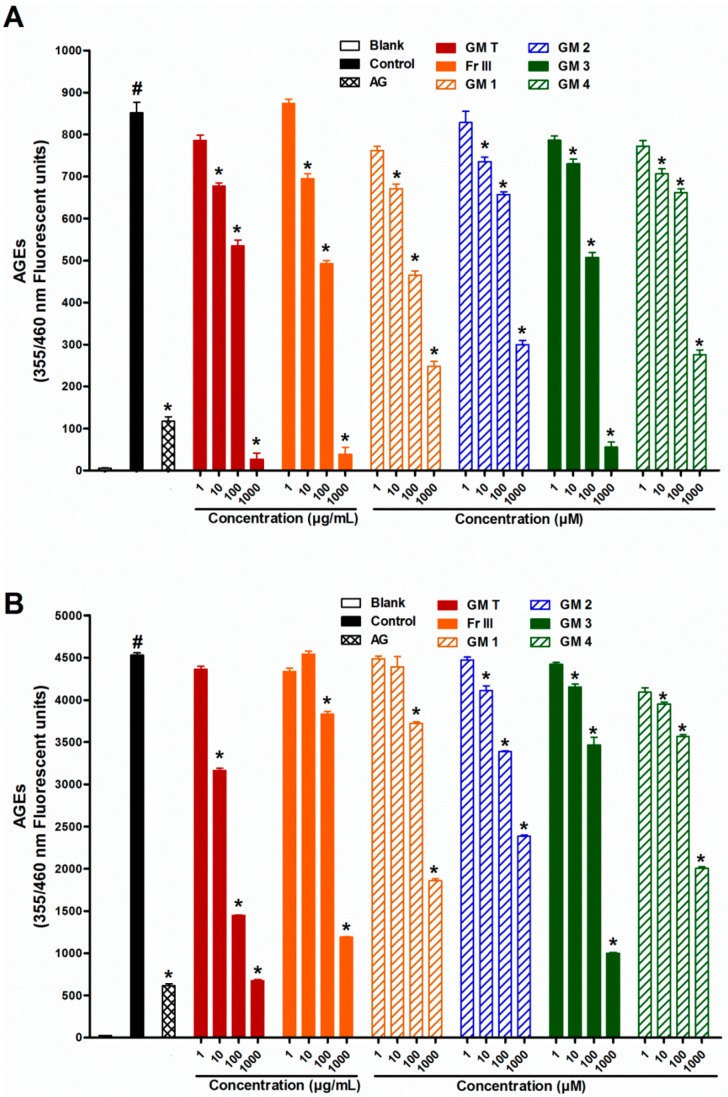
Effect of GMT, Fr III and isolated compounds on the formation of fluorescent AGEs when BSA incubated with with glucose (**A**) or ribose (**B**) at week 4. Blank is a reaction mixture including BSA and glucose or ribose kept at –20 °C while control is the same reaction mixture but incubated at 37 °C. AG was used as standard anti AGEs drug. Results are expressed as mean ± SEM (*n* = 3). ^#^ *p* < 0.05 when compared to Blank, * *p* < 0.05 when compared to Control.

**Figure 2:**
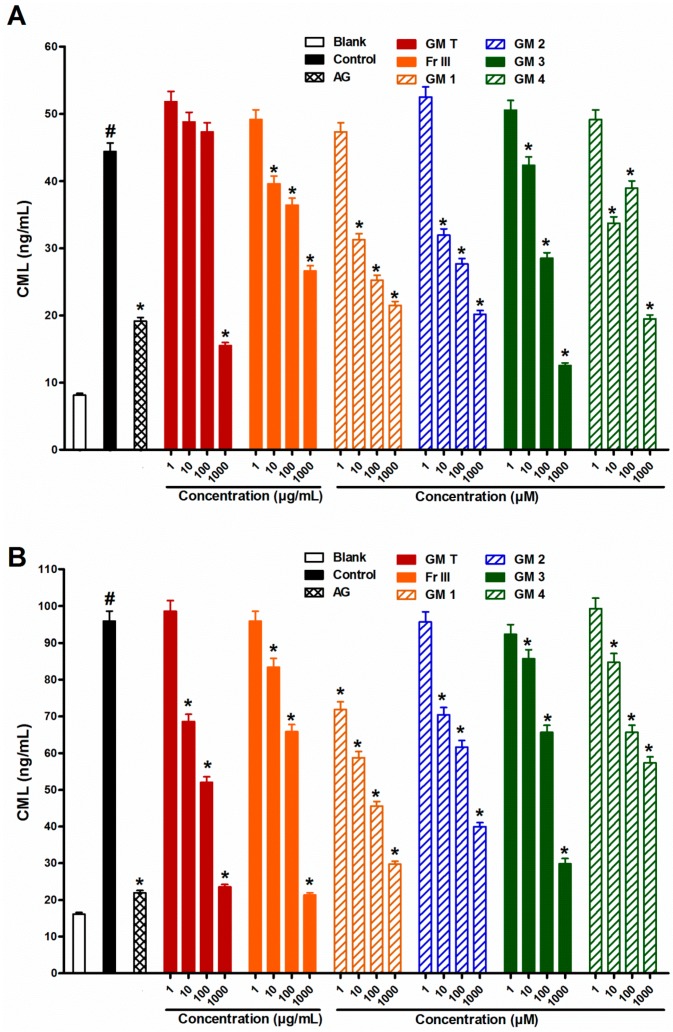
Effect of GMT, Fr III and isolated compounds on non-fluorescence AGEs level (*N*_ε_-CML) when BSA incubated with glucose (**A**), and ribose (**B**) at week 4. Blank is a reaction mixture including BSA and glucose or ribose kept at −20 °C while control is the same reaction mixture but incubated at 37 °C. AG was used as standard anti AGEs drug. Results are expressed as mean ± SEM (*n* = 3). ^#^ *p* < 0.05 when compared to Blank, * *p* < 0.05 when compared to Control.

Incubation of bovine serum albumin (BSA) with glucose or ribose for four weeks significantly increased the formation of fluorescent (Figure 1) and non-fluorescent (CML) AGEs compared with the corresponding blank (Figure 2).

The reaction between monosaccharides and proteins generates irreversible heterogeneous products, called advanced glycation endproducts (AGEs) which is clinically used as an indicator for short-term control of blood sugars in diabetic patients [20]. Formation of AGEs was higher in ribose than glucose, which is in agreement with previous studies that reported higher ability of ribose than glucose to exhibit protein cross-linking. Moreover, previous studies revealed that glycating ability of d-glucose was less than that of d-ribose [21]. The higher glycating capability of d-ribose is explained by its planar structure causing the unstable aldofuranose ring to react with the amino groups [22]. AGE formation was significantly inhibited by aminoguanidine (1000 µM), the standard AGE inhibitor.

**Figure 3:**
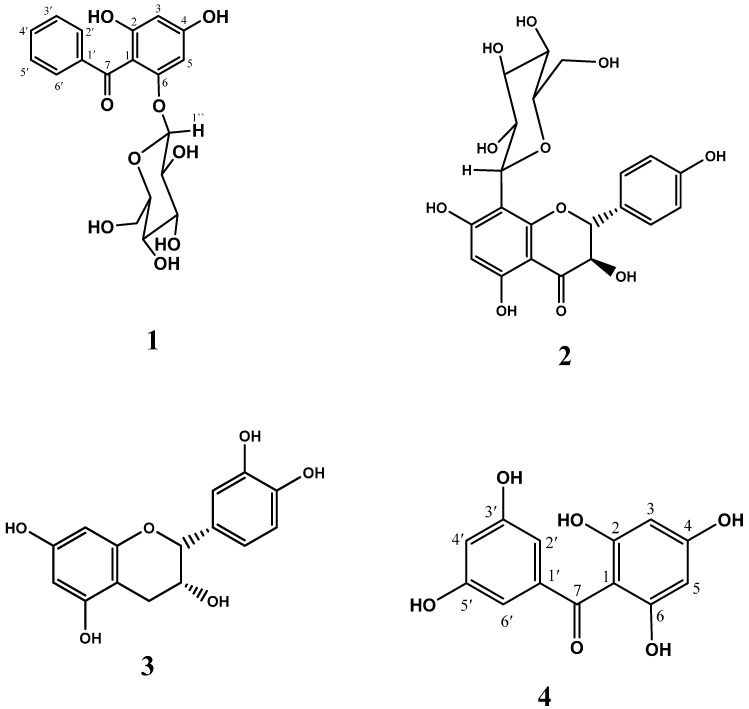
Chemical structures of compounds **1**–**4** isolated from *Garcinia mangostana*.

Addition of GMT (10–1000 µg/mL) to the reaction mixture inhibited fluorescent and non-fluorescent AGE formation in a dose dependent manner in both glucose and ribose (Figure 1 and Figure 2). The bioassay-guided fractionation of GMT revealed that fraction III (Fr III, 10–1000 µg/mL) showed potent anti-AGE formation activity upon addition to the reaction mixture. It significantly inhibited fluorescent (93% and 77% inhibition at 1000 µg/mL for glucose and ribose, respectively) and non-fluorescent AGEs formation (88% and 75% inhibition at 1000 µg/mL for glucose and ribose, respectively) in a dose dependent manner (Figure 1 and Figure 2). The other fractions showed weak inhibition of AGE formation in the case of glucose (33% and 17% inhibition at 1000 µg/mL for fractions I and II, respectively) Phytochemical investigation of Fr III allowed the isolation of four bioactive metabolites (Figure 3), which were identified based on comparison of their spectral data (^1^H, ^13^C, HSQC, HMBC) with previously published data and confirmed through co-chromatography with authentic samples (Figures S1–S12 and Table S1). These isolated compounds were identified as garcimangosone D (**1**) [23], aromadendrin-8-*C*-glucopyranoside (**2**) [24], epicatechin (**3**) [25], and 2,3′,4,5′,6-pentahydroxybenzophenone (**4**) [26]. The isolated compounds are polyphenolic, suggesting possible alleviation of apopotic mechanisms. It has been widely accepted that plant-derived polyphenolics affect mitochondria functionality in healthy and pathological *in vitro* and *in vivo* models. On the other hand, mitochondria-related apoptotic mechanims are implicated in the development of a wide range of diseases, including metabolic, cardiovascular, degenerative and hyperproliferative pathologies [27]. 

Including compounds **1**–**4** in the reaction mixture at concentrations of (1–1000 µM) significantly inhibited fluorescent and non-fluorescent AGE formation in a dose dependent manner in both glucose and ribose (Figure 1 and Figure 2). Compound **3** (epicatechin) was the most potent; it inhibited fluorescent AGEs by 94% and 80% while compound **2** was the least effective (64% and 46% inhibition) for glucose and ribose, respectively. Meanwhile, compound **3** showed also high inhibition of formation of non-fluorescent AGEs (89% and 82%) for glucose and ribose, respectively, while compound **1** was the least effective in the case of glucose (62%) and compound **4** was the least effective in the case of ribose (43%).

Several major mechanisms by which polyphenols block the carbonyl group in reducing sugars and break the cross-linking structure in the formed AGEs have recently been proposed for antiglycation activity [7]. Our findings are consistent with previous reports indicating that glucose- and ribose-induced glycation increased protein oxidation. The activity of compounds **2** and **3** against glycation was, at least partly, due to their antioxidant properties. Previous reports confirmed inhibitory activity of flavonoid nucleus on glycation. Luteolin, qurcetin and rutin exhibited significant inhibitory activity on HbA_1C_ formation. Meanwhile; luteolin and rutin produced more significant inhibitory effect on methylglyoxal-mediated protein modification, while luteolin was found to be potent inhibitor of both the AGEs formation and subsequent cross-linking of protein [28]. The anti-AGEs effect of epicatechin (**3**) was previously reported, whereby it was able to break preformed glycated human serum albumin *in vitro* as well as reduce AGE accumulation in retinas *in vivo* in a dose dependent manner [29]. Moreover it was able to inhibit pentosidine formation [17]. In this work the activity of **2** was first to be reported, while aromadendrin (the aglycon of **2**) was previously reported to inhibit the formation of AGEs [30]. Moreover, few reports are available on anti-glycation activity of benzophenones (**1** and **4**). Recently, only garcimangosone D (**1**) showed strong activity as a pentosidine formation inhibitor [17]. Anti-AGE formation activity could occur through inhibition of radical chain reaction by its radical scavenging activity [31]. 

Herein, it was reported for the first time that inhibition of AGE formation by phenolic compounds was more pronounced on ribose than glucose. The anti-oxidant effect of phenolics may react by some way to decrease the reaction rate between ribose (a pentose) and amino acids than that of glucose (a hexose). The ability of polyphenols to block the carbonyl group in reducing sugars and break the cross-linking structure in the formed AGEs may be easier in ribose due to its planar structure [7,22]. The explanation of these results need further studies to correlate the higher affinity of pentose to form AGEs and its failure to form these products in the presence of phenolic compounds. 

### 2.2. Effect of GMT, Bioactive Fraction and Active Metabolites on Fructosamine (An Amadori Product) 

**Figure 4:**
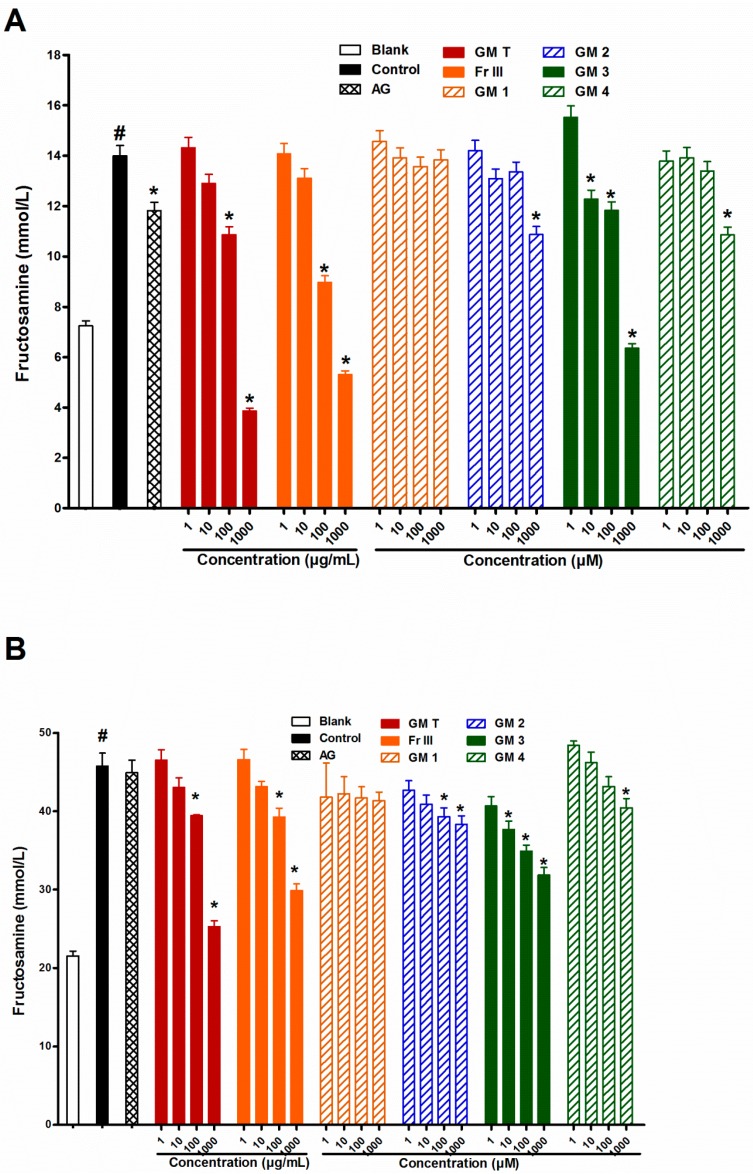
Effect of GMT, Fr III and isolated compounds on fructosamine (An amadori product) level when BSA incubated with glucose (**A**), and ribose (**B**) at week 4. Blank is a reaction mixture including BSA and glucose or ribose kept at −20 °C while control is the same reaction mixture but incubated at 37 °C. AG was used as standard anti AGEs drug. Results are expressed as mean ± SEM (*n* = 3). ^#^ *p* < 0.05 when compared to Blank, * *p* < 0.05 when compared to Control.

In search for the level of action, addition of GMT, Fr III (1–1000 µg/mL) to the reaction mixture inhibited fructosamine formation in a dose dependent manner after incubating BSA with glucose or ribose. Compounds **2**–**4** at 1000 µM were able to significantly inhibit fructosamine formation, whereas metabolite **3**, epicatechin, strongly inhibited fructosamine formation (100% for glucose and 56% for ribose) (Figure 4). This points to the responsibility of the tested bioactive compounds for early inhibition of AGEs at the Amadori product level. Being metal chelators and/or radical scavengers, some flavonoids (**2** and **3**) have the ability to suppress the levels of fructosamines [32]. Catechins were reported to inhibit the formation of AGEs by their radical scavenging activity, but metal ions and free radicals do not participate in the early stage (*i.e.*, in the formation of fructosamines). It is noteworthy that, benzophenone glycoside **1** didn’t display any activity in this assay, while benzophenone aglycone **4** showed activity only at a concentration of 1000 µM. 

### 2.3. Effect of GMT, Bioactive Fraction and Active Metabolites on Protein Aggregation 

**Figure 5:**
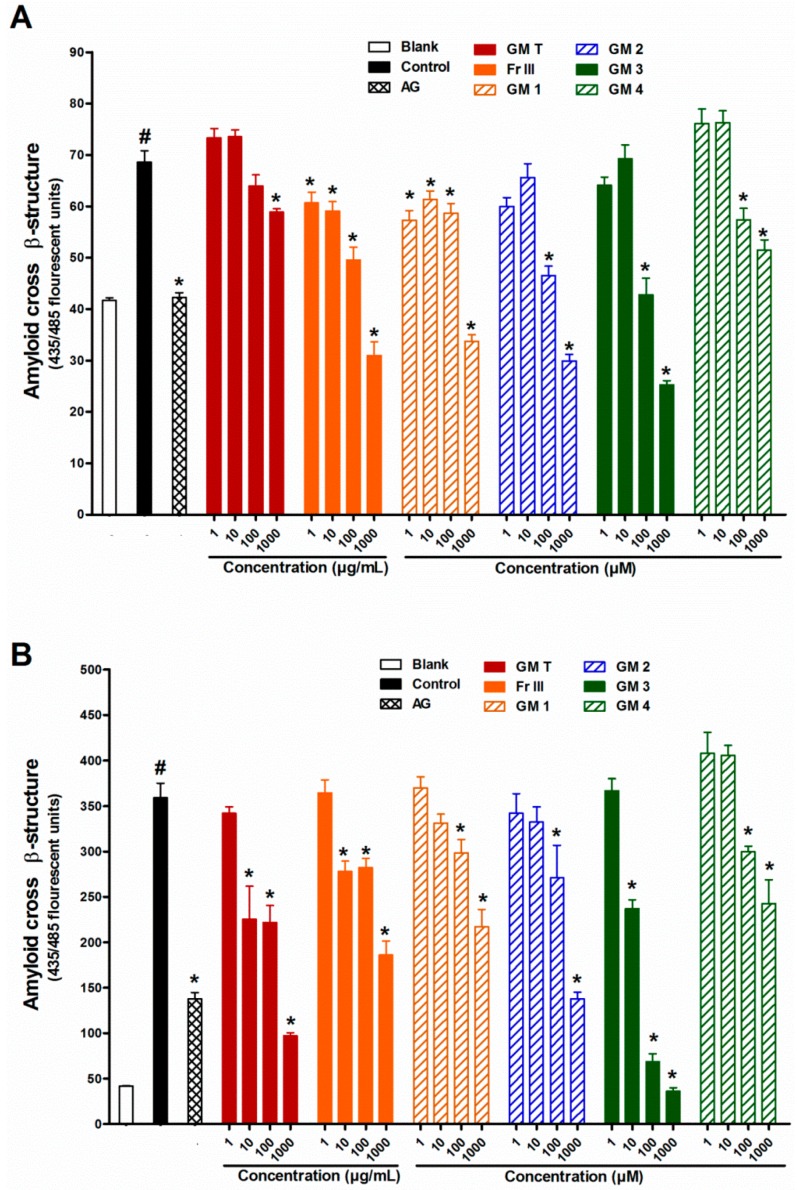
Effect of GMT, Fr III and isolated compounds on amyloid cross β-structure levels when BSA incubated with glucose (**A**), and ribose (**B**) at week 4. Blank is a reaction mixture including BSA and glucose or ribose kept at −20 °C while control is the same reaction mixture but incubated at 37 °C. AG was used as standard anti AGEs drug. Results are expressed as mean ± SEM (*n* = 3). ^#^ *p* < 0.05 when compared to Blank, * *p* < 0.05 when compared to Control.

To investigate the different stages of AGE formation, the effect of GMT, bioactive fraction and isolated metabolites **1**–**4** on protein aggregation was studied. Addition of the GMT or Fr III (1–1000 µg/mL) to the reaction mixture inhibited the amyloid cross β-structure formation due to incubation of BSA with glucose or ribose in a dose dependent manner. All the bioactive metabolites were able to significantly inhibit amyloid cross β-structure formation, while metabolite **3**, epicatechin, was the most potent (150% for glucose and 100% for ribose, Figure 5). On the other hand, compound **4** was the least effective (60% and 36% inhibition) for glucose and ribose, respectively. 

This indicates that compounds **1**–**4** are responsible for inhibition of AGE formation at the protein aggregation level. Protein glycation is believed to be a key mechanism to accelerate the formation of protein aggregation and amyloid cross β-structures leading to altered protein structure and stability [33,34]. The long-term accumulation of amyloid cross β-structures in tissues and organs is linked to the progression of pancreatic islet amyloidosis which directly destroys β-cell and impairs insulin secretion [35,36]. The present findings demonstrate that GMT, bioactive fraction and compounds **1**–**4** reduce the formation of amyloid cross β-structures in BSA. This beneficial effect might help reduce the risk of developing diabetes complications.

### 2.4. Effect of GMT, Bioactive Fraction and Active Metabolites on Protein Thiol Group 

**Figure 6:**
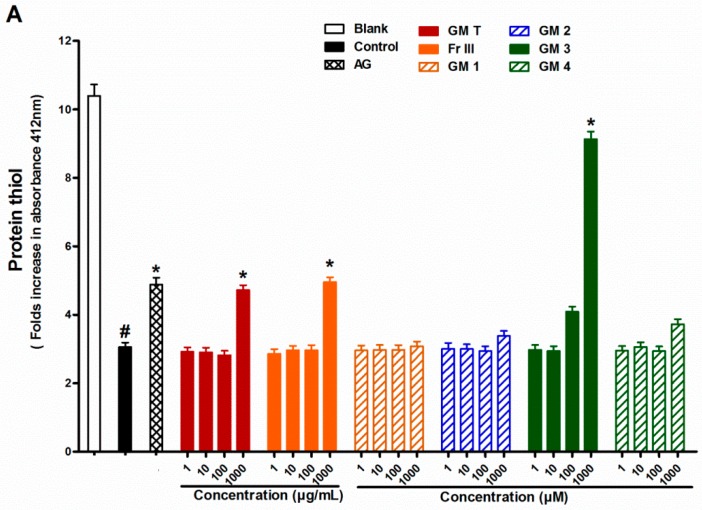

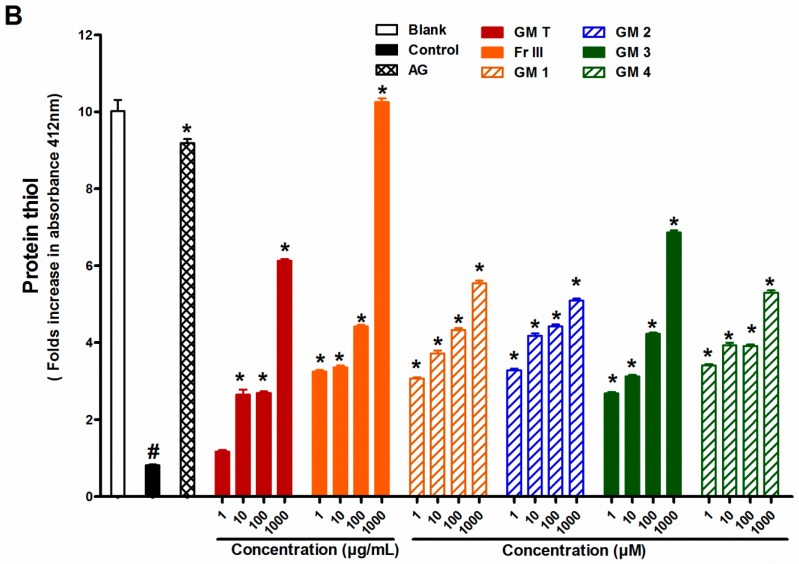
Effect of GMT, Fr III and isolated compounds on protein thiol levels when BSA incubated with glucose (**A**), and ribose (**B**) at week 4. Blank is a reaction mixture including BSA and glucose or ribose kept at −20 °C while control is the same reaction mixture but incubated at 37 °C. AG was used as standard anti AGEs drug. Results are expressed as mean ± SEM (*n* = 3). ^#^ *p* < 0.05 when compared to Blank, * *p* < 0.05 when compared to Control.

In search for the possible mechanism of action, the effect of GMT, bioactive fraction and active metabolites on protein thiol group was investigated. Addition of the GMT or Fr III (1–1000 µg/mL) to the reaction mixture inhibited the depletion in protein thiols caused by incubating BSA with glucose or ribose. All the bioactive metabolites were able to significantly alleviate protein thiol depletion in the case of ribose. Compound **3** showed the highest activity (66% inhibition), while compounds **2** and **4** were the least effective (44% inhibition). On contrary only metabolite **3**, epicatechin, prevented protein thiol depletion in the case of glucose (Figure 6).

Thiol groups of proteins are a general target to determine the protein oxidation during glycation [37,38]. Cysteine and methionine are particularly prone to oxidative attack by free radical species such as superoxide and hydroxyl radicals. The direct oxidation of amino acid (Lys, Arg, Thr) or secondary reaction of amino acid residues (Cys and His) with reactive carbonyl compounds can produce the formation of protein carbonyl content (PCO) derivatives [39]. Hence, PCO is commonly used as a marker for protein oxidative damage [40]. The formation of AGEs is a multifactorial process. One of the mechanisms of AGE formation and protein damage is through the production of reactive oxygen species (ROS). It is well established that protein glycation continually generates superoxide anions from early glycation products that include 1,2- and 2,3-enolization of the Schiff’s base and oxidation of the enolate anion [41,42]. In addition, the Amadori product or Schiff's base undergoes fragmentation through reactive oxygen species-mediated reactions to generate short-chain carbohydrate intermediates, which alter lysine and arginine residues to produce AGEs. According to the formation of non-fluorescent and non-crosslinking structures so far, hydroxyl radicals generated by Fenton reactions between Fe^2+^ and Amadori product-derived endogenous H_2_O_2_ causes oxidative cleavage of Amadori compounds into *N*_ε_-CML [43]. This mechanism is involved in the defense against free radical attack on proteins [7]. Like many polyphenols, compounds **1**–**4** exhibit antioxidant activity by scavenging superoxide and hydroxyl radicals, suggesting that inhibition of protein glycation and AGE formation may be related to their antioxidant activity [44,45]. The results showed that GMT, bioactive fraction and active metabolites can be as effective as AG for suppression of protein glycation. Unfortunately, AG was discontinued in Phase III clinical trials possibly due to its toxicity [46]. Based on the current knowledge, GMT, bioactive fraction and active metabolites could be considered as promising inhibitor of AGE formation. 

The ability of flavonoids **2** and **4** to protect thiol groups was previously reported. Flavonoids decreased glycation through an increase in the antioxidant component dependent on the levels and activities of thiol-containing proteins such as glutathione peroxidase [32]. Meanwhile, no reports on the activity of benzophenone on thiol group protection were found but its effect may be through inhibition of radical chain reaction by its radical scavenging activity [31].

## 3. Experimental Section

### 3.1. Chemicals

Bovine serum albumin (BSA, fraction V), d-ribose, d-glucose, and 2,4-dinitrophenylhydrazine (DNPH), nitroblue tetrazolium (NBT), 1-deoxy-1-morpholino-D-fructose (1-DMF), aminoguanidine hydrochloride, thioflavin T and 5,5′-dithiobis(2-nitrobenzoic acid) (DTNB) were purchased from Sigma-Aldrich Co. (St. Louis, MO, USA). OxiSelect™ *N*_ε_-(carboxymethyl) lysine (*N*_ε_-CML) ELISA kit was obtained from Cell Biolabs (San Diego, CA, USA). All other chemicals and solvents used in this study were analytical grade.

### 3.2. General Experimental Procedures

TLC analysis was performed on pre-coated TLC plates with silica gel 60 F_254_ (Merck, Darmstadt, Germany). Column chromatographic separations were performed on silica gel 60 (70–230 mesh, Merck), Diaion HP-20 (Merck) and polyamide 6 for column chromatography (Merck). 1D and 2D NMR spectra were recorded on a DRX-400 MHz Ultrashield spectrometer (Bruker BioSpin, Billerica, MA, USA) using CD_3_OD as solvent, with TMS as the internal reference. 

### 3.3. Plant Material

The fruits of *G. mangostana* were purchased from the local market in Saudi Arabia, in December, 2014. The plant material was authenticated by Associate Professor Emad Al-Sharif (Plant Ecology, Dept. of Biology, Faculty of Science & Arts, Khulais, King Abdulaziz University, Kingdom of Saudi Arabia). A herbarium specimen (No. GM1424) was prepared and deposited at the Herbarium of the Faculty of Pharmacy, King Abdulaziz University.

### 3.4. Extraction and Isolation

The air-dried fruit hulls of *G. mangostana* (500 g) were extracted with MeOH (1 L × 3) at room temperature using an Ultraturrax, until exhaustion and the combined extracts were evaporated under vacuum. The methanol extract (GMT, 20 g) was suspended in water (200 mL) and partitioned with chloroform (300 mL × 3) to produce a chloroform-soluble fraction (4 g, fraction I). The remaining solution was applied on a column of Diaion HP-20 (6 cm × 110 cm, 250 g) and eluted with H_2_O (1 L), followed by 50% MeOH in H_2_O (2 L), and finally 100% MeOH (2 L). The eluates were evaporated under vacuum to give 2.5 g (Fraction II), 7 g (Fraction III), and 4 g (Fraction IV) of dry residue, respectively. Most of Fraction III (6 g) was applied to a polyamide column (6 × 100 cm, 250 g), and eluted with H_2_O followed by a gradient of H_2_O/MeOH mixtures to pure MeOH. On the basis of TLC with the use of UV light, 5% AlCl_3_, 1% FeCl_3_, or anisaldehyde-H_2_SO_4_ spray reagents for detection, similar fractions were pooled together to yield three collective fractions (A–C).

Fraction A (10 and 20 % MeOH) was subjected to silica gel column (25 cm × 2 cm, 50 g) and eluted with a CHCl_3_–MeOH mixture (9.5:0.5, *v*/*v*) to give compounds **1** (14 mg) and **2** (125 mg). Fraction B (40% and 50% MeOH) was subjected to silica gel column (25 cm × 2 cm, 50 g) and eluted with a CHCl_3_–MeOH mixture (9:1, *v*/*v*) to give compounds **3** (150 mg). Finally, Fraction C (70%–100% MeOH) was subjected to silica gel column (25 cm × 2 cm, 50 g) and eluted with a CHCl_3_–MeOH mixture (8:2, *v*/*v*) to give compounds **4** (30 mg).

The purity of isolated compounds was detected by HPLC system consisted of an Agilent 1200 system, a solvent delivery module, a quaternary pump, an autosampler, and a diode-array detector (DAD) (Agilent Technology, Baden-Württemberg, Germany), using an Agilent Zorbax Extend-C18 column (150 mm length × 4.6 mm, i.d., 5 μm).

### 3.5. In Vitro Glycation of Bovine Serum Albumin (BSA) Induced by Monosaccharides

Glycation of BSA was done according to a previous method with minor modifications [47]. Briefly, 10 mg/mL BSA was incubated with 500 mM glucose and 100 mM ribose in 100 mM phosphate buffered-saline (pH 7.4) containing 0.02% sodium azide at 37 °C (optimum temperature for reaction BSA and the sugar) for four weeks in the absence (Control) or presence of GMT, Fr III, compounds or AG (a positive control). Complete reaction mixture including BSA and glucose or ribose in PBS plus azide was stored at −20 °C (to stop the reaction between BSA and sugars) and used as Blank (Our modification to the method). BSA, glucose and ribose were used at concentrations reported to form clear significant AGEs *in vitro* in previous studies . Before incubation, GMT and Fr III (final concentration: 1–1000 µg/mL) and isolated compounds **GM1**–**4** (final concentration: 1–1000 µM) and AG (final concentration: 1000 µM) were added into the reaction mixtures. A final concentration of 1% dimethylsulfoxide (DMSO) was used as solvent for the study. 

### 3.6. Determination of Advanced Glycation End-Products (AGEs) 

The formation of fluorescent AGEs was measured as previously described [48,49,50] using monochromator SpectraMax^®^ M3 plate reader (Molecular Devices, Sunnyvale, CA, USA). The fluorescence intensity was measured at excitation set at 485 nm and emission set at 525 nm. The concentration of non-fluorescent AGEs (*N*_ε_-(carboxymethyl) lysine, *N*_ε_-CML), a major non-fluorescent AGE structure, was measured by using an enzyme linked immunosorbant assay (ELISA) kit according to the manufacturer’s protocol. The concentration of *N*_ε_-CML was calculated by using a *N*_ε_-CML-BSA standard curve. 

### 3.7. Determination of Fructosamine (An Amadori Products) 

The level of the Amadori product fructosamine was measured as fructosamine in the same reaction mixtures in which AGEs were measured by using nitroblue-tetrazolium (NBT) dye according to a previous method with minor modifications [51]. Glycated BSA was incubated with 0.5 mM NBT in 100 mM carbonate buffer (pH 10.4) at 37 °C. The absorbance was measured at 530 nm every 3 minutes. The level of fructosamine was calculated using the different absorption at the time point of 9 and 12 min (our modification to the method), and compared to 1-deoxy-1-morpholino-d-fructose (1-DMF) as the standard. 

### 3.8. Determination of Protein Aggregation 

Amyloid cross β-structure, a common marker for protein aggregation was measured in the same reaction mixtures in which AGEs were measured using the thioflavin T assay according to a previous method with minor modifications [52]. Glycated BSA was incubated with 3.2 µM thioflavin T in 10 mM phosphate buffered-saline (pH 7.4) at room temperature for 60 min (our modification to the method). The fluorescence intensity was measured at an excitation wavelength of 435 nm and emission wavelength of 485 nm. 

### 3.9. Determination of Protein Thiol Group 

Protein thiol group was measured in the same reaction mixtures in which AGEs were measured according to Ellman’s assay with minor modifications [53]. Glycated BSA was incubated with 5 mM 5,5′-dithiobis(2-nitrobenzoic acid) (DTNB) in 10 mM phosphate buffered-saline (pH 7.4) at room temperature for 15 min and the absorbance measured at 412 nm before and after addition of DTNB. The results were expressed as folds increase in absorbance (our modification to the method). 

### 3.10. Statistical Analysis 

Values are expressed as mean ± SEM. Statistical analysis was performed by the one way analysis of variance (ANOVA) followed by Dunnett’s post hoc test using GraphPad Prism software, version 5.00 (GraphPad Software Inc., La Jolla, CA, USA). 

## 4. Conclusions

*G. mangostana* was evaluated for its inhibitory effects against two monosaccharide-mediated protein glycation reactions and oxidative damage of BSA. *G. mangostana* markedly inhibited protein glycation and oxidative damage in BSA induced by glucose and ribose. The isolated bioactive compounds that belong to the flavonoid (**2** and **3**) and benzophenone (**1** and **4**) classes of compounds decreased the formation of fluorescent, non-fluorescent AGEs, and fructosamine associated with the reduction of protein aggregation and protein carbonyl content. They also prevented the loss of protein thiol groups. Anti-AGEs formation activity could be due to inhibition of radical chain reactions by their radical scavenging activity. Given the ability of *G. mangostana* to inhibit glycation reactions with monosaccharides, we conclude that the use of GMT will prevent accumulation of AGEs and result in reduction of one of most important diabetic complications.

## Supplementary Materials

The following are available online at http://www.mdpi.com/1420-3049/21/2/251/s1. Figures S1–S8: ^1^H- and ^13^C-spectra and HPLC conditions for purity detection of compounds **1**–**4**, Table S1: NMR spectral data of compounds **1**–**4** (CD_3_OD, 400 and 100, MHz).
