# Supplementary material for: Phenolics from Garcinia mangostana Inhibit Advanced Glycation Endproducts Formation: Effect on Amadori Products, Cross-Linked Structures and Protein Thiols
Source: Molecules. 2016 Feb 22;21(2):251. doi: 10.3390/molecules21020251 (PMC6273600; doi:10.3390/molecules21020251)
Supplement: Supplementary file 1 [file molecules-21-00251-s001.pdf]

Molecules **2016**, *21*, 251; doi:10.3390/molecules21020251

Supplementary Materials: Phenolics from *Garcinia mangostana* Inhibit Advanced Glycation Endproducts Formation: Effect on Amadori Products, Cross-Linked Structures and Protein Thiols

Hossam M. Abdallah [1,2,*], Hany El-Bassossy [3,4], Gamal A. Mohamed [1,5], Ali M. El-halawany [1,2], Khalid Z. Alshali [6] and Zainy M. Banjar [7]

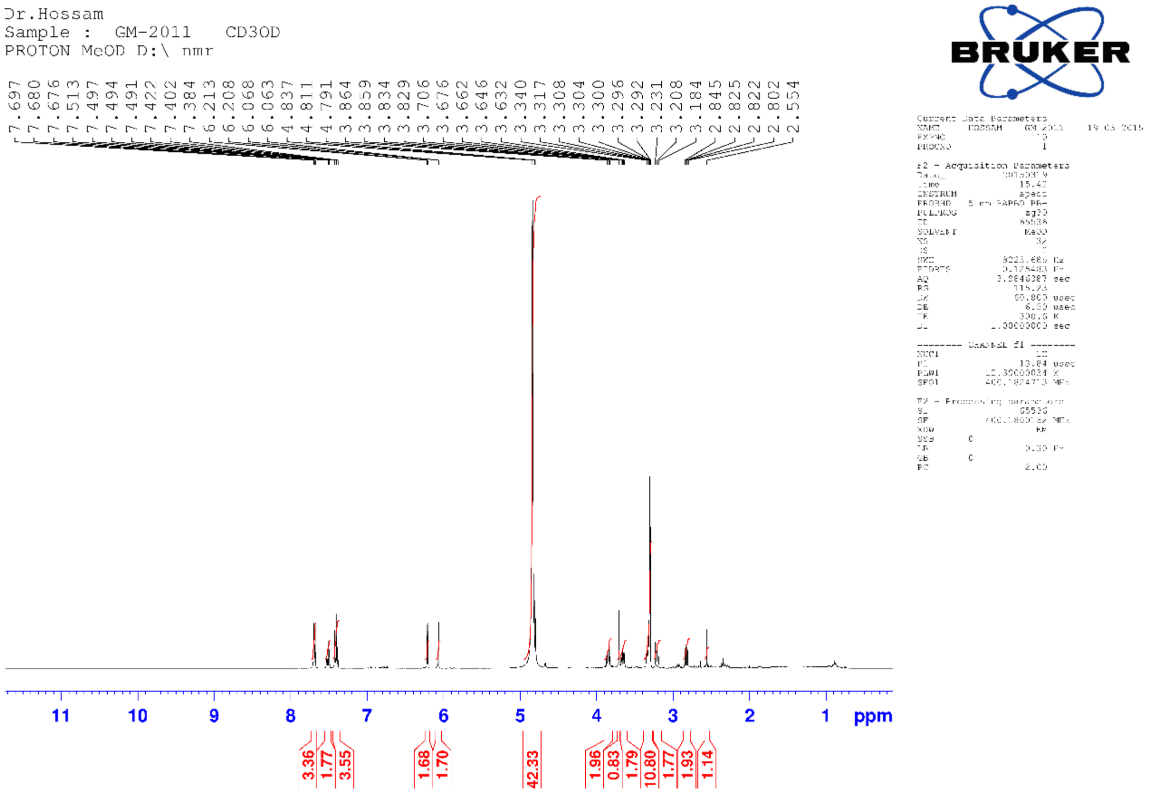

Figure S1. ^{1}H-NMR of compound **1**.

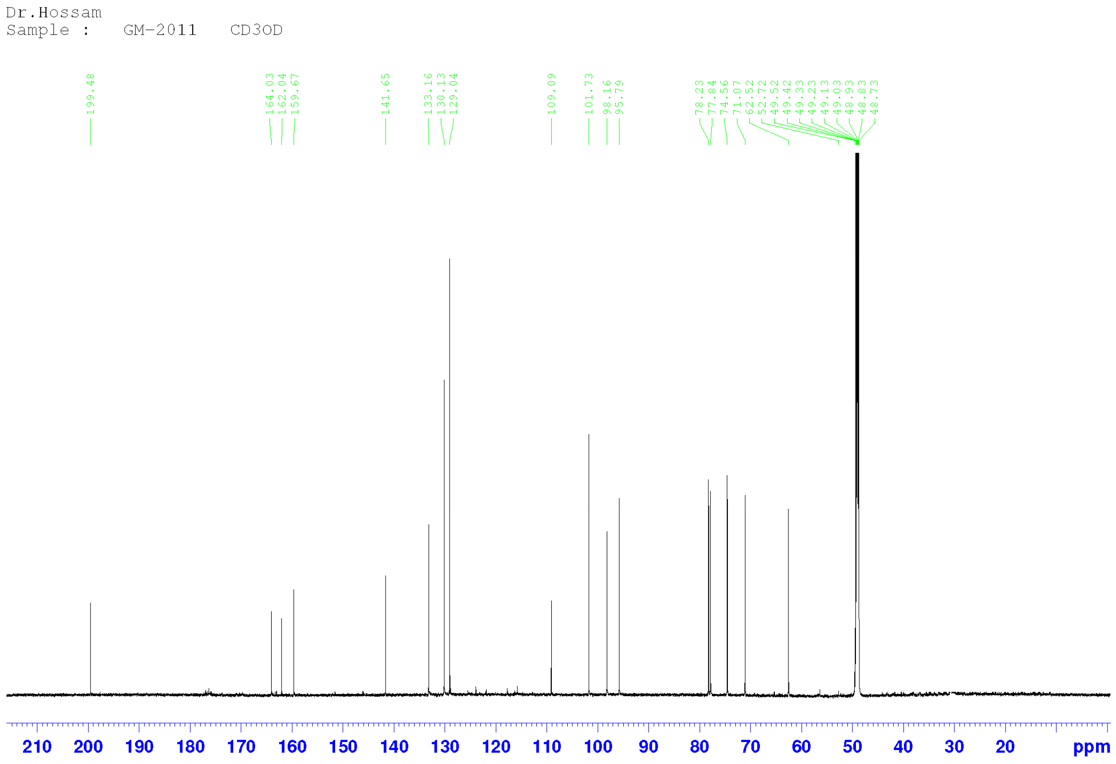

Figure S2. ^{13}C-NMR of compound **1**.

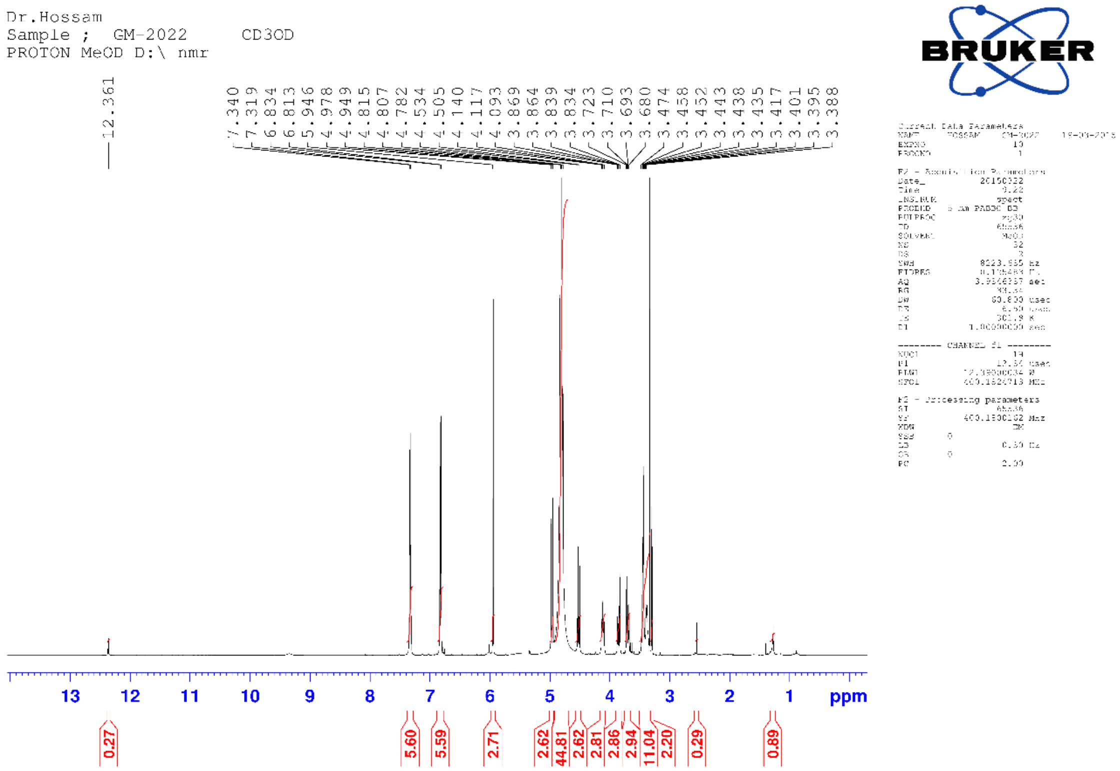

Figure S3. ^{1}H-NMR of compound **2**.

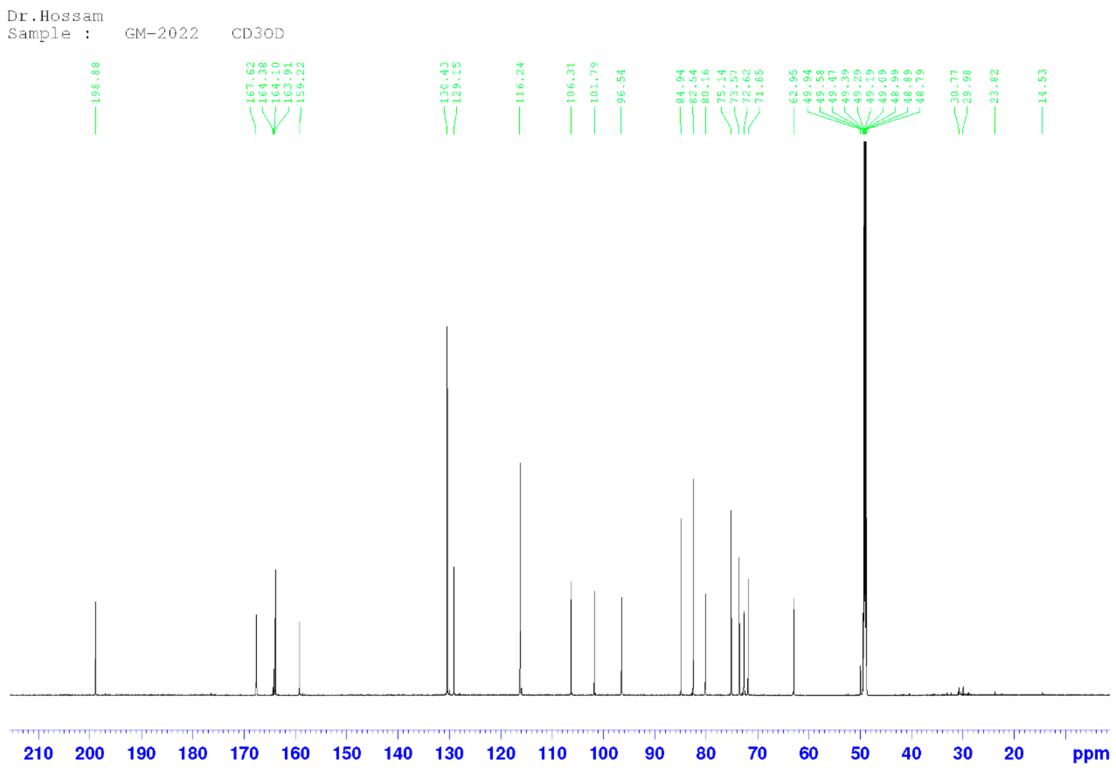

Figure S4. ^{13}C-NMR of compound **2**.

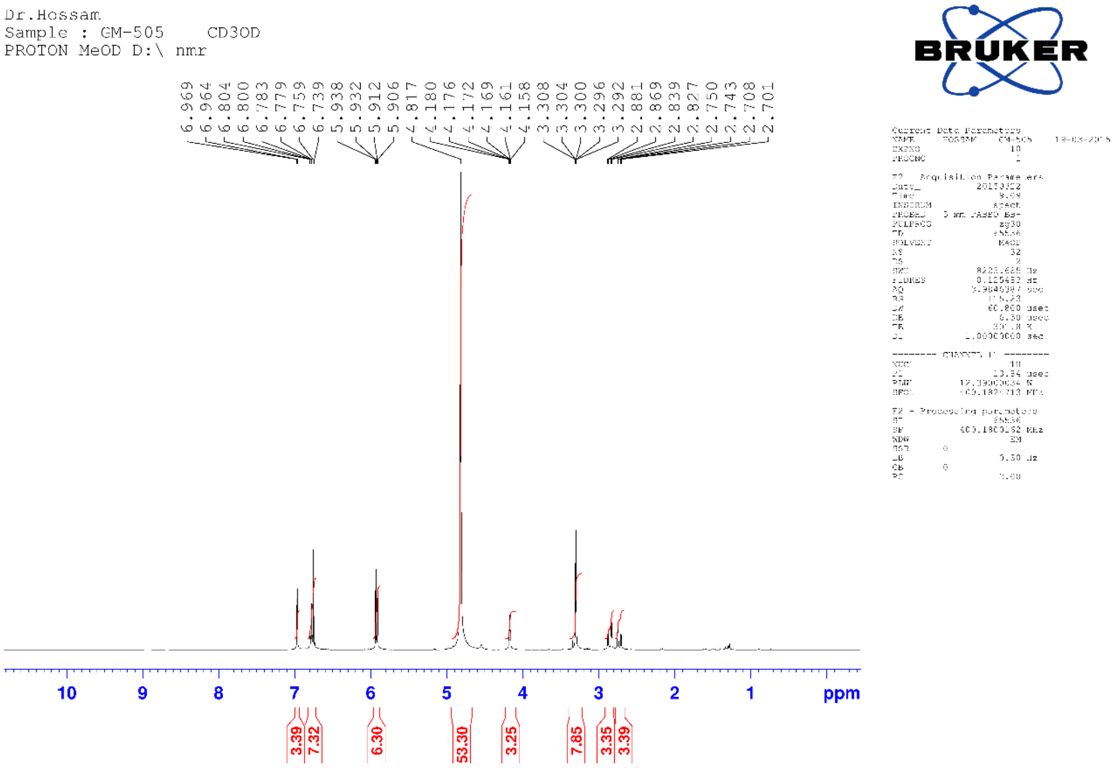

Figure S5. ^{1}H-NMR of compound **3**.

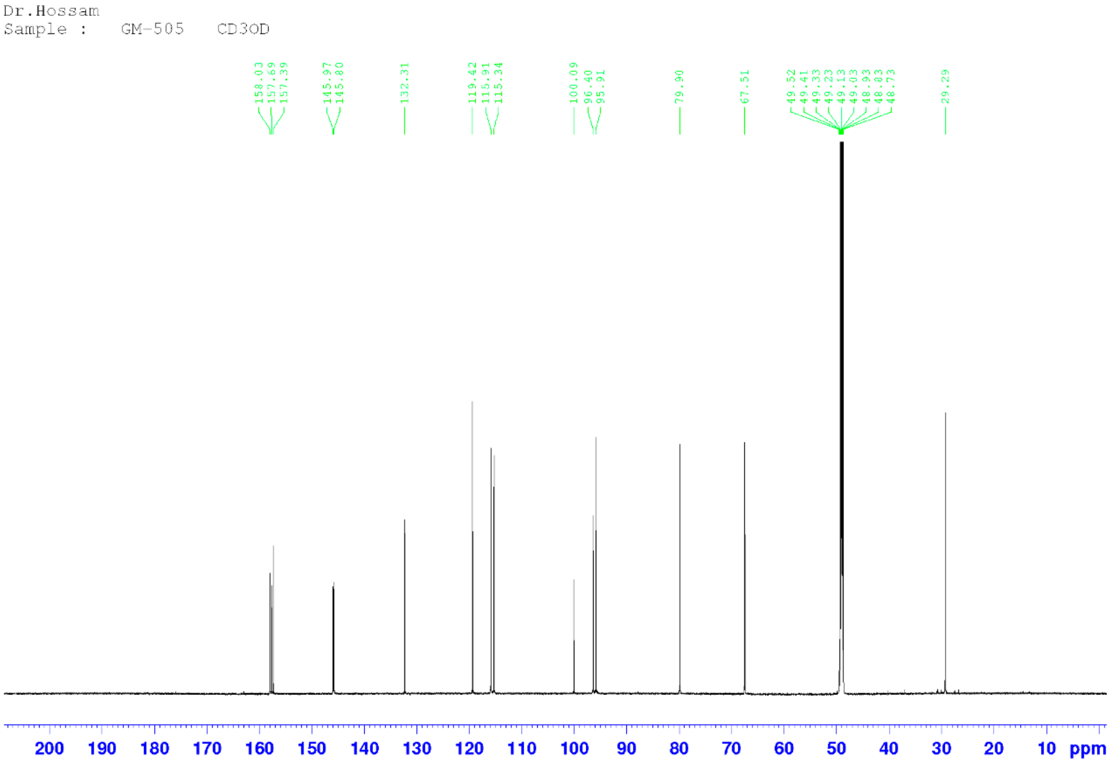

Figure S6. ^{13}C-NMR of compound **3**.

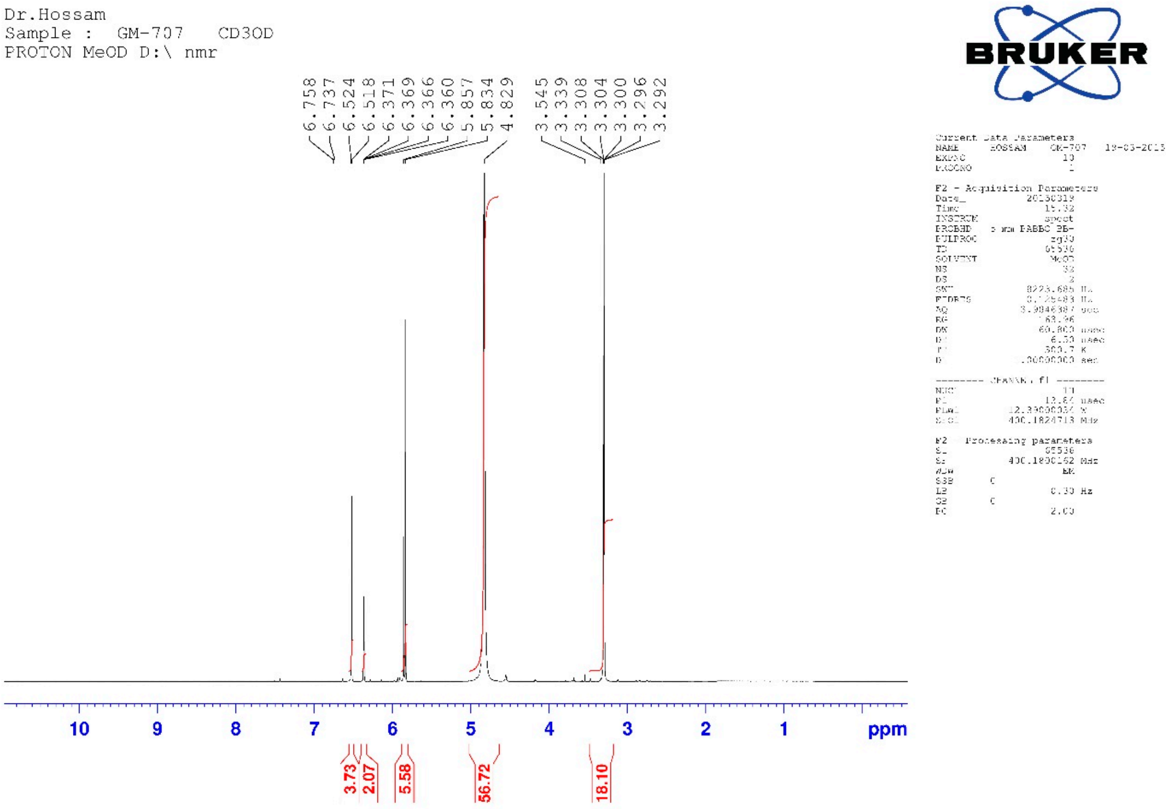

Figure S7. ^{1}H-NMR of compound **4**.

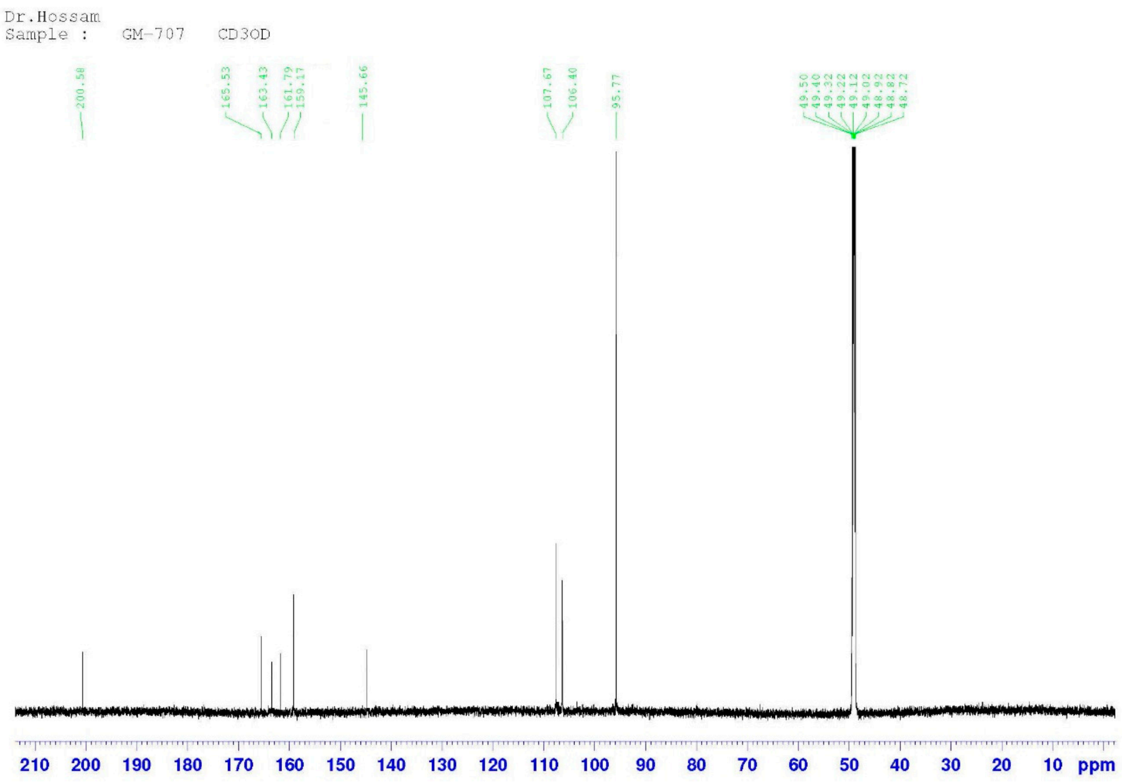

Figure S8. ^{13}C-NMR of compound **4**.

HPLC Condition

Instrument

The HPLC system consisted of an Agilent 1200 system, a solvent delivery module, a quaternary pump, an autosampler, a diode-array detector (DAD), and a column compartment (Agilent Technology, Waldbronn, Baden-Württemberg , Germany).

Chromatographic Conditions

Column: Agilent Zorbax Extend-C18 column (150 mm × 4.6 mm, i.d. 5 μm). column Temperature 40 °C.

Mobile phase: H_2O has 0.1% phosphoric acid (solvent A) and Methanol has 0.1%phosphoric acid (solvent B).

The gradiant elution process began at 95% solvent B for 20 min, followed by a linear increase in solvent A to 40% at 40 min. Lastly, solvent B decreased back to 5% at 50 min.

Detector: UV at λ = 280 nm

Flow rate: 0.5 mL/min.

Injection volume: 5 μL

Nota bene: Peak identification was based on the comparison of retention times and UV spectra of the standard and compounds.

Table S1. NMR spectral data of compounds **1–4** (CD_3OD, 400 and 100, MHz).

No	1		2		3		4	
	δ_H (mult., *J* (Hz))	δ_C (mult.)	δ_H (mult., *J* (Hz))	δ_C (mult.)	δ_H (mult., *J* (Hz))	δ_C (mult.)	δ_H (mult., *J* (Hz))	δ_C (mult.)
1	-	109.1 (C)	-	-	-	-	-	106.4 (C)
2	-	162.0 (C)	4.96 d (12.4)	84.9 (CH)	4.82 s	79.9 (CH)	-	161.8 (C)
3	6.06 d (2.0)	98.2 (CH)	4.52 d (12.4)	73.6 (CH)	4.71 m	67.5 (CH)	5.83 s (2.0)	95.7 (CH)
4	-	164.0 (C)	-	198.9 (C)	2.87 dd (16.6, 4.8)<br>2.73 dd (16.6, 2.8)	29.3 (CH_2)	-	165.5 (C)
5	6.24 d (2.0)	95.8 (CH)	-	163.9 (C)	-	157.4 (C)	5.83 s (2.0)	95.7 (CH)
6	-	159.7 (C)	5.95 s	96.5 (CH)	5.91 d (2.4)	96.4 (CH)	-	163.4 (C)
7	-	199.5 (C)	-	167.6 (C)		158.0 (C)	-	200.6 (C)
8			-	105.6 (C)	5.94 d (2.4)	95.9 (CH)		
9	-	-	-	164.1 (C)	-	157.7 (C)	-	-
10	-	-	-	101.8 (C)	-	100.1 (C)	-	-
1′	-	141.7 (C)	-	122.2 (C)	-	132.3 (C)	-	145.7 (C)
2′	7.69 m	130.1 (CH)	7.33 d (8.4)	130.4 (CH)	6.97 d (2.0)	115.4 (CH)	6.73 s	106.4 (CH)
3′	7.38 m	129.0 (CH)	6.82 d (8.4)	116.2 (CH)	-	145.8 (C)	-	159.2 (C)
4′	7.49 m	133.2 (CH)	-	159.2 (C)	-	146.0 (C)	6.52 m	107.7 (CH)
5′	7.38 m	129.0 (CH)	6.82 d (8.4)	116.2 (CH)	6.75 d (8.4)	115.9 (CH)		159.2 (CH)
6′	7.69 m	130.1 (CH)	7.33 d (8.4)	130.4 (CH)	6.80 dd (8.4, 2.0)	119.4 (CH)	6.73 s	106.4 (CH)
1′′	4.85 d (7.8)	101.7 (CH)	4.78 d (7.6)	75.1 (CH_2)	-	-		
2′′	2.80 m	74.5 (CH)	4.12 m	72.6 (CH)	-	-		
3′′	3.23 m	77.8 (CH)	3.46 m	80.2 (CH)	-	-	-	-
4′′	3.18 m	71.1 (CH)	3.43 m	71.9 (CH)	-	-	-	-
5′′	3.4 m	78.2 (CH)	3.30 m	82.5 (CH)	-	-	-	-
6′′	3.82 dd (12.0, 2.0)<br>3.63 dd (12.0, 5.6)	62.5 (CH_2)	3.84 dd (12.0, 2.0)<br>3.70 dd (12.0, 5.2)	63.0 (CH_2)				
